# Is there a relationship between serum vitamin D and semen parameters? A cross-sectional sample of the Iranian infertile men

**DOI:** 10.1186/s12610-021-00147-3

**Published:** 2021-12-02

**Authors:** Hossein Hajianfar, Elham Karimi, Negar Mollaghasemi, Sheyda Rezaei, Arman Arab

**Affiliations:** 1 Food Safety Research Center (salt), Semnan University of Medical Sciences, Semnan, Iran; 2 Community Health Research Center, Isfahan (khorasgan) Branch, Islamic Azad University, Isfahan, Iran; 3 Nutrition Research Center, School of Nutrition and Food Sciences, Shiraz University of Medical Sciences, Shiraz, Iran; 4 Department of Clinical Nutrition, School of Nutrition and Food Science, Food Security Research Center, Isfahan University of Medical Sciences, Isfahan, Iran; 5 Research Development Center, Arash Women’s Hospital, Tehran University of Medical Sciences, Tehran, Iran; 6 Student Research Committee, School of Nutrition and Food Sciences, Semnan University of Medical Sciences, Semnan, Iran; 7 Department of Community Nutrition, School of Nutrition and Food Science, Food Security Research Center, Isfahan University of Medical Sciences, Isfahan, Iran

**Keywords:** Vitamin D, Semen quality, Fertility, Male, Vitamine D, Qualité du sperme, Fertilité, Homme

## Abstract

**Background:**

Recent studies suggest that serum vitamin D may be associated with semen parameters. In the present cross-sectional study, we attempted to investigate the association between serum vitamin D levels and semen parameters among Iranian sub-fertile men.

**Results:**

A total of 350 infertile men recruited for this cross-sectional study using a simple random sampling method with a mean age of 34.77 years old, body mass index of 26.67 kg/m^2^, serum vitamin D of 20.17 ng/ml, semen volume of 3.82 mL, sperm count of 44.48 (10^6^/mL), sperm total motility of 38.10 %, and morphologically normal sperm of 7.0 %. After controlling for potential confounders, serum vitamin D was positively associated with semen volume (β = 0.63, 95 % CI: 0.06, 1.20), sperm count (β = 14.40, 95 % CI: 4.56, 24.25), sperm total motility (β = 18.12, 95 % CI: 12.37, 23.86), and sperm normal morphology (β = 1.95, 95 % CI: 1.07, 2.83).

**Conclusions:**

The present findings suggest that higher serum vitamin D levels are positively associated with higher semen volume, sperm count, sperm total motility, and normal morphology rate. These findings, however, do not specify a cause-and-effect relationship, and there is a need for further research in this area to understand whether vitamin D supplementation can improve semen parameters.

## Résumé

**Contexte:**

Des études récentes suggèrent que la vitamine D sérique peut être associée aux paramètres spermatiques. Dans la présente étude transversale, nous avons tenté d’étudier l’association entre les taux sériques de vitamine D et les paramètres spermatiques chez des hommes Iraniens inféconds.

**Résultats:**

Un total de 350 hommes inféconds ont été recrutés pour cette étude transversale à l’aide d’une méthode d’échantillonnage aléatoire simple, avec un âge moyen de 34,8 ans, un indice de masse corporelle de 26,7 kg/m2, une vitamine D sérique de 20,2 ng/ml, un volume de sperme de 3,8 mL, un nombre de spermatozoïdes de 44,5 (106/mL), une motilité totale des spermatozoïdes de 38 %, et des spermatozoïdes morphologiquement normaux de 7,0 %. Après contrôle des facteurs potentiels de confusion, la vitamine D sérique a été positivement associée au volume de sperme (β = 0,63, IC à 95 % : 0,06, 1,20), au nombre de spermatozoïdes (β = 14,40, IC à 95 % : 4,56, 24,25), à la motilité totale des spermatozoïdes (β = 18,12, IC à 95 % : 12,37, 23,86) et à la morphologie normale des spermatozoïdes (β = 1,95, IC à 95 % : 1,07, 2,83).

**Conclusion:**

Les présents résultats suggèrent que des taux sériques plus élevés de vitamine D sont positivement associés à un volume de sperme, un nombre de spermatozoïdes, une motilité totale des spermatozoïdes et un taux de morphologie normale plus élevés. Cependant, ces résultats ne spécifient pas de relation de cause à effet, et il est nécessaire de poursuivre les recherches dans ce domaine pour comprendre si la supplémentation en vitamine D peut améliorer les paramètres du sperme.

## Introduction

Infertility is characterized as the inability to achieve pregnancy after 12 months of regular unprotected sexual intercourse which affects 2.5–15 % of couples worldwide and correlating to at least 30 million infertile men globally [1]. Lots of men diagnosed with infertility are experiencing different semen abnormalities such as low sperm count, low sperm motility, and impaired sperm function that resulting in incapability to fertilize an oocyte in the absence of explicit etiologic factors [2]. For decades, human semen quality has been degraded due to potential factors including air pollutants or toxicants, obesity, smoking, drinking, electromagnetic waves from cell phones, and dietary factors [3–9]. Studies regarding male infertility covered a wide range and aimed to explore the mechanism of the disease at molecular and biochemical levels [10]. In this regard, vitamin D has gained lots of attention in recent years with a role that goes beyond calcium/phosphorous homeostasis and bone health [11].

Vitamin D, a fat-soluble nutrient, is primarily synthesized in the skin from cholesterol via a process dependent on sun exposure [12]. It has been reported that low levels of vitamin D have a bearing on an increased risk in various health issues [13, 14]. Recent clinical and experimental studies have proposed that vitamin D is crucial for male reproductive functions, and vitamin D receptors (VDRs) and metabolizing enzymes are expressed in the genital tract and germ cells of males [11]. However, the issue of whether vitamin D is associated with male reproductive biology and fertility status has not been resolved extensively. A recent systematic review and meta-analysis suggested that vitamin D may play a substantial role in the sexual health of men [10]. However, most of the present knowledge regarding vitamin D and infertility comes from non-Middle Eastern countries with contradictory results [15–17]. Moreover, previous documents which were carried out in Iran also failed to provide a significant association between vitamin D and male fertility [18, 19].

Therefore, it seems that further investigation regarding the possible association between vitamin D and semen quality is needed in Iran as a Middle Eastern country with a better methodology and larger sample size. In the present cross-sectional study, we attempted to investigate the association between serum vitamin D levels and semen parameters among Iranian sub-fertile men. Findings will be used to notify public health programming and improve therapeutic intervention among men with infertility problems.

## Methods and materials

### Study settings and population

**Fig. 1:**
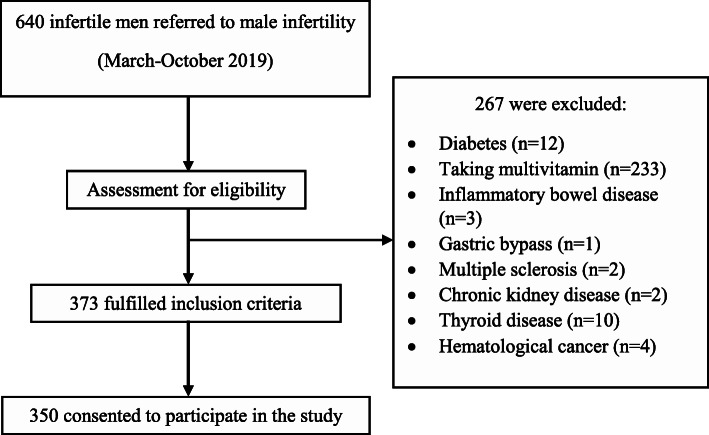
Flow chart of the participants selection process

The present study was conducted in accordance with the ethical criteria of the Declaration of Helsinki (1964) and was also approved by the Ethics Committee of the Semnan University of Medical Sciences (IR.SEMUMS.REC.1397.277). Moreover, written informed consent was obtained from all of the participants. From March to October 2019, 640 infertile adult males who were referred to the Isfahan Fertility and Infertility center, a referral center in Isfahan, Iran, for semen analysis due to male infertility were consecutively evaluated. Finally, 350 subfertile men, aged 20–50 years recruited for this cross-sectional study using a simple random sampling method. All of the included males were from couples who had failed to conceive after 1 year of unprotected and regular sexual intercourse, with the exception of female cause of infertility. Participants were excluded if they had chronic disease (i.e., type 2 diabetes, liver, thyroid, and renal disease), urogenital infections, malabsorption syndromes, malignancy, osteometabolic disorders. Patients who taking multivitamins were also excluded. Figure 1 shows the participants selection process.

### Semen collection and analysis

Semen collection was carried out after 2–5 days of sexual abstinence and analyzed within 1 h of ejaculation, on the basis of World Health Organization (WHO) guidelines [20]. Semen analysis was done by an expert technician using the CASA system [SCATM, Microptic, Version 4.2, Barcelona, Spain]. CASA system included a phase-contrast microscope (Nikon™ Eclipse E-200, Tokyo, Japan) with a heat plate. Using weighting, semen volume was estimated. To assess the percentage of sperm total motility, 10 mL of well-mixed semen sample were examined under a microscope at ×400 magnification while semen was kept at 37° C in the heat plate. The average of two assessment was reported as the final percentage of sperm total motility. Following the semen sample dilution in a solution of 0.4 % (v/v) formaldehyde and 0.6 mol/L NaHCO3, only spermatozoa with tail were counted. At last, smears were prepared for the assessment of sperm morphology using Papanicolaou stained according to strict criteria [21].

### Blood sampling and vitamin D analysis

A fasting venous blood sample was obtained from each participant on the morning of semen collection. The serum was separated by centrifugation at ×3500 rpm for 10 min and subsequently stored at -80 °C for vitamin D analysis. Vitamin D analysis was carried out using the Electro-chemiluminescence immunoassay (ECLIA) kits (manufactured by Roche Diagnostics GmbH, Mannheim, Germany) with 7.8 % and 10.7 % intra- and inter-assay CV, respectively. Patients were then categorized as deficient [25(OH)D < 10 ng/mL], insufficient [10 ng/mL ≤ 25(OH)D ≥ 20 ng/mL], and sufficient [25(OH)D > 20 ng/mL] according to the previous reports among Iranian population [18].

### Dietary intake assessment

The dietary intake of participants during the previous year was examined using a validated semi-quantitative 168-item food frequency questionnaire (FFQ) [22–24] through a face-to-face interview. This questionnaire consisted of a list of food items alongside serving sizes for each. The subjects were asked to report their intake for each food item on a daily, weekly, or monthly basis. Then the reported serving size converted to gram using the household measures [25]. All data were analyzed by Nutritionist IV software (First Databank, Hearst Corp., San Bruno, CA, USA).

### Assessment of other variables

Through a face-to-face interview, demographic information including age, educational status, job, smoking, alcohol consumption, and diagnosis of varicocele was collected. Weight was measured using a digital scale (Omron BF511, Omron Corp., Kyoto, Japan) and height was estimated by an upstretched tape to the nearest 100 g and 0.1 cm, respectively. The Body Mass Index (BMI) was calculated via weight in kg and height in m using related formula.

### Statistical analysis

All of the analysis was done using the SPSS software version 26 (IBM Corp., Armonk, NY, USA). P-values less than 0.05 were considered statistically significant. Before data analysis, the normality of continuous variables was assessed via the Q-Q plot, skewness statistic, and histogram chart. The qualitative and quantitative variables were expressed as number (percent) and mean ± standard error (SE), respectively. The differences of continuous variables across categories of serum vitamin D were assessed by the analysis of variance (ANOVA). The distribution of categorical variables across categories of serum vitamin D was examined using the Chi-square test. The relationship between serum vitamin D and semen parameters was assessed via a multiple linear regression analysis which was performed in different models and presented as beta (β) estimates with the corresponding 95 % confidence intervals (CIs). In the first model, we adjusted for age. Further adjustment was made for educational status, smoking, alcohol consumption, job, and varicocele. In the final model, total energy intake was controlled as surrogate data to obtain a diet-independent association between serum vitamin D and semen parameters.

## Result

The demographic information, semen parameters, and dietary intake of participants are shown in Table 1. Overall, 350 infertile men make up our study population with a mean (SE) age of 34.77 (0.42) years, a weight of 83.18 (0.73) kg, a height of 176.51 (0.40) cm, a BMI of 26.67 (0.20) kg/m^2^, serum vitamin D of 20.17 (0.55) ng/ml, semen volume of 3.82 (0.10) mL, sperm count of 44.48 (1.89) 10^6^/mL, sperm total motility of 38.10 (1.13) %, and morphologically normal sperm of 7.0 (0.17) %. Individuals in the highest category of serum vitamin D had higher semen volume, sperm count, sperm total motility, and morphologically normal sperm, and were less likely to be azoospermic compared to those in the lowest category. Moreover, males with insufficient vitamin D status had a higher age compared to those with deficient vitamin D status. No significant difference was observed in terms of other demographic and dietary intake variables (all *P* values > 0.05).

**Table 1:** General characteristics of the study population across categories of serum vitamin D

Variable	Categories of serum vitamin D			*P*-value†
	Deficient (< 10 ng/ml)	Insufficient (10–20 ng/ml)	Sufficient (> 20 ng/ml)	
**N**	67	119	164	
***Demographic information***				
** Age (year)**	32.57 ± 0.98	35.85 ± 0.83	34.89 ± 0.53	0.025
** Weight (kg)**	81.59 ± 1.57	82.29 ± 1.14	84.49 ± 1.17	0.240
** Height (cm)**	174.75 ± 0.94	176.96 ± 0.65	176.90 ± 0.60	0.104
** BMI (kg/m2)**	26.68 ± 0.42	26.25 ± 0.30	26.98 ± 0.32	0.272
** Serum vitamin D (ng/mL)**	7.20 ± 0.21	14.78 ± 0.28	29.37 ± 0.51	< 0.001
** University education**	36 (53.7)	61 (51.3)	80 (48.8)	0.480
** Employee**	26 (38.8)	44 (37.0)	52 (31.7)	0.149
** Current smoker**	28 (41.8)	48 (40.3)	65 (39.6)	0.767
** Having varicocele**	23 (34.3)	57 (47.9)	70 (42.7)	0.443
***Semen parameters***				
** Semen volume (mL)**	3.51 ± 0.27	3.51 ± 0.13	4.18 ± 0.17	0.009
** Sperm count (×10**^**6**^**/mL)**	36.44 ± 4.75	43.77 ± 3.08	48.28 ± 2.72	0.068
** Sperm total motility (%)**	24.96 ± 2.93	38.36 ± 1.82	43.28 ± 1.64	< 0.001
** Normal morphology (%)**	5.54 ± 0.47	6.76 ± 0.28	7.77 ± 0.22	< 0.001
** Azoospermia**				
** Yes**	39 (58.2)	57 (47.9)	72 (43.9)	0.058
** No**	28 (41.8)	62 (52.1)	92 (56.1)	
***Dietary intake***				
** Total energy (kcal/d)**	1757.32 ± 72.78	1684.90 ± 47.97	1695.50 ± 40.47	0.652
** Carbohydrate (g/d)**	246.19 ± 3.64	243.99 ± 2.56	249.66 ± 2.48	0.286
** Protein (g/d)**	61.89 ± 1.44	61.62 ± 1.07	62.98 ± 0.90	0.597
** Total fat (g/d)**	56.60 ± 1.58	57.82 ± 1.05	54.85 ± 1.01	0.139
** Total fiber (g/d)**	17.55 ± 0.74	17.95 ± 0.53	18.31 ± 0.46	0.665

Data are presented as mean ± standard error or number (% within categories of serum vitamin D)

Continuous variables calculated by analysis of variance (ANOVA) and categorical variables by Chi-square test

*P* < 0.05 was considered statistically significant

*BMI* Body Mass Index

The beta estimates and the corresponding 95 % CIs for the association between serum vitamin D and semen parameters are presented in Table 2. In the crude model, serum vitamin D was positively associated with semen volume (β = 0.67, 95 % CI: 0.10, 1.24) in those in the top category of serum vitamin D compared to the lowest category. This association remained also significant after controlling for age, education, smoking, job status, varicocele, total energy intake, and BMI (β = 0.63, 95 % CI: 0.06, 1.20). The higher serum vitamin D levels were significantly associated with higher sperm count in males with sufficient vitamin D status compared to deficient ones, either before (β = 11.83, 95 % CI: 1.84, 21.82) or after adjustment for potential confounders (β = 14.40, 95 % CI: 4.56, 24.25). After controlling for age, education, smoking, job status, varicocele, total energy intake, and BMI, an increase of 18.12 % in the sperm total motility (95 % CI: 12.37, 23.86) and 1.95 % in the sperm normal morphology (95 % CI: 1.07, 2.83) was observed when the mean serum vitamin D increased from 7.20 ng/mL (deficient category) to 29.37 ng/mL (sufficient category).

**Table 2:** Beta (β) and 95 % confidence interval for semen-quality parameters according to categories of serum vitamin D

	Categories of serum vitamin D			
	Deficient (< 10 ng/ml)	Insufficient (10–20 ng/ml)	Sufficient (> 20 ng/ml)	P trend
Semen volume (mL)				
**Crude**	Ref	0.004 (-0.59, 0.60)	0.67 (0.10, 1.24)	0.006
**Model 1**	Ref	-0.05 (-0.66, 0.55)	0.63 (0.05, 1.20)	0.008
**Model 2**	Ref	-0.006 (-0.61, 0.60)	0.62 (0.04, 1.19)	0.010
**Model 3**	Ref	0.03 (-0.57, 0.64)	0.63 (0.06, 1.20)	0.010
Sperm count (10^6^/mL)				
**Crude**	Ref	7.32 (-3.19, 17.85)	11.83 (1.84, 21.82)	0.021
**Model 1**	Ref	8.57 (-2.02, 19.16)	12.71 (2.70, 22.72)	0.015
**Model 2**	Ref	10.03 (-0.49, 20.56)	13.23 (3.32, 23.13)	0.013
**Model 3**	Ref	9.99 (-0.43, 20.42)	14.40 (4.56, 24.25)	0.005
Sperm total motility (%)				
**Crude**	Ref	13.39 (7.36, 19.43)	18.31 (12.59, 24.04)	< 0.001
**Model 1**	Ref	13.57 (7.48, 19.66)	18.44 (12.68, 24.20)	< 0.001
**Model 2**	Ref	13.82 (7.72, 19.92)	18.32 (12.58, 24.06)	< 0.001
**Model 3**	Ref	13.60 (7.51, 19.69)	18.12 (12.37, 23.86)	< 0.001
Normal morphology (%)				
**Crude**	Ref	1.21 (0.26, 2.16)	2.22 (1.31, 3.12)	< 0.001
**Model 1**	Ref	0.93 (-0.005, 1.87)	2.02 (1.13, 2.91)	< 0.001
**Model 2**	Ref	1.02 (0.09, 1.95)	2.00 (1.12, 2.88)	< 0.001
**Model 3**	Ref	1.05 (0.12, 1.98)	1.95 (1.07, 2.83)	< 0.001

Data are presented as β (95 % confidence interval)

†*P* < 0.05 was considered statistically significant

Crude: Unadjusted

Model 1: Adjusted for age

Model 2: Model 1 + educational status, smoking, alcohol consumption, job, and varicocele

Model 3: Model 2 + body mass index and total energy intake

## Discussion

Vitamin D is a fat-soluble micronutrient that has been mostly known for its roles in calcium/phosphate homeostasis and bone health [10]. Recently, the role of vitamin D in the pathogenesis of infertility and impaired semen parameters has been considered in various studies [15, 16, 18, 26]. Increasing studies have shown conflicting relationships between vitamin D and semen parameters that some studies showing a significant association between serum vitamin D and semen parameters [16, 27], while others did not support such an association [19, 28].

In the current cross-sectional study, we examined the association between serum vitamin D levels and semen parameters among the Iranian sample of infertile men. We found that lower serum vitamin D was significantly associated with impaired semen parameters including semen volume, sperm count, percentage of sperm total motility, and sperm morphology.

Previous studies have reported the prevalence of vitamin D deficiency from 12.4 to 48.6 % [29]. The positive effect of vitamin D on human spermatozoa has been shown in vitro studies [15, 30], although its exact mechanisms are not yet known. Nevertheless, VDR is found in male reproductive tissues such as the human testis, ejaculatory tract, and mature spermatozoa [31], and this indicates the putative role of vitamin D in human reproduction. Recent cross-sectional studies have shown conflicting results on the association between serum vitamin D levels and semen parameters [15, 26, 32]. The present study found a significantly positive association between serum vitamin D levels and sperm count and normal morphology after controlling for potential confounders. This finding was also verified by previous studies [32–34]; conversely, few studies have reported contradictory results [15, 17, 18, 26, 35]. These conflicts may be due to the dependence of sperm quality and count on other parameters such as the optimal levels of related enzymes, hormones, and antioxidants [36]. Moreover, differences in ethnicity [37], genetic variations [38], the concentration of air pollutants [39], and other confounders may be the source of contradictory results as compared to previous studies. Our findings also revealed that vitamin D was significantly associated with percentage of sperm total motility, which is in line with some previous reports [15, 16] and inconsistent with others [18, 26, 32, 40]. Moreover, a recent meta-analysis also indicated a significant relationship between vitamin D and sperm motility and progressive motility, but not for other semen parameters [10]. Literature indicates that vitamin D raises intracellular calcium levels and motility of sperm; it also brings the acrosome reaction in mature spermatozoa, and there was a positive association between serum 25(OH)D levels and sperm motility [34]. Nevertheless, there is still a gap in knowledge about the exact mechanism of action of vitamin D on sperm parameters; therefore, more studies are suggested to confirm this relationship. Recently, increasing evidence points to the role of vitamin D in various tissues and organs, especially those in reproductive function and spermatogenesis. However, the mechanisms by which vitamin D affects male reproduction are unclear [41]. Vitamin D can be effective in optimizing sperm function directly and it may also be influenced indirectly through calcium homeostasis [18]. Vitamin D exerts its physiological effects through VDR, which is abundant in the reproductive system including spermatozoa [42]. Evidence showed the presence of VDR and vitamin D metabolism enzymes in adult spermatozoa suggesting the functional role of vitamin D in the reproductive system. Furthermore, vitamin D directly affects germ cells and the cells lining the reproductive tract [43, 44]. Vitamin D deficiency reduces sperm quality; moreover, there is suppression of testicular germ cell production and a decline in mature seminiferous tubules percentage in mice [45]. Also, vitamin D deficiency reduces the enzymes for testosterone synthesis and, consequently, serum, and testicular testosterone levels were decreased [45]. Further studies indicated that vitamin D could increase the content of calcium in the neck and head of spermatozoa, which might be a probable link between vitamin D and sperm motility and also, acrosome activity [46].

### Limitations of the study

One of the limitations of this study is that we didn’t estimate calcium and phosphorus levels, parathyroid, and reproductive hormones status including LH, FSH, and testosterone levels, as several studies suggest an association between vitamin D and these hormones. Also, our study neither noted the seasonal differences in vitamin D levels among participants. Moreover, we did not look at the genetic polymorphism of VDR or vitamin D binding protein. Also, the current study used a cross-sectional design which precludes us to draw a causal relationship. Moreover, there was no clinical assessment regarding the presence of andrological pathologies. Since, these pathologies may be responsible for an altered spermatogenesis, their potential distribution in the different category of vitamin D could induce a bias in the results. The abstinence delay was not assessed as a confounder factor; therefore, it could induce a bias regarding semen volume and sperm count. Additionally, absence of the genetic checkup (i.e., karyotype and microdeletions of the Y Chromosome) for oligozoospermia and azoospermia was another limitation of this study.

## Conclusions

The present findings suggest that higher serum vitamin D levels are positively associated with higher semen volume, sperm count, percentage of sperm total motility, and normal morphology rate. These findings, however, do not specify a cause-and-effect relationship, and there is a need for further research in this area to understand whether vitamin D supplementation can improve semen parameters and also to discover the mechanisms that mediate this association.

## Data Availability

The data that support the findings of this study are available from the corresponding author upon reasonable request.

## Abbreviations

ANOVA: Analysis of variance
BMI: Body Mass Index
CI: Confidence interval
ECLIA: Electro-chemiluminescence immunoassay
FFQ: Food frequency questionnaire
SE: Standard error
VDR: Vitamin D receptors
WHO: World Health Organization
